# Owner Willingness to Temporarily Store Firearms With Firearm Retailers and Law Enforcement Agencies

**DOI:** 10.1001/jamanetworkopen.2025.21921

**Published:** 2025-07-16

**Authors:** Jennifer Paruk, Erin Wright-Kelly, Leslie Barnard, Michael Anestis

**Affiliations:** 1 New Jersey Gun Violence Research Center, Rutgers University School of Public Health, Piscataway; 2 Firearm Injury Prevention Initiative, School of Medicine, University of Colorado Anschutz Medical Campus, Aurora; 3 Injury and Violence Prevention Center, University of Colorado Anschutz Medical Campus, Aurora

## Abstract

**Importance:**

Voluntary and temporary firearm storage outside the home in times of heightened risk, including where unauthorized access may be possible, may reduce firearm deaths. Firearm retailers and law enforcement agencies may provide this storage, but little is known regarding variability among firearm owners and their households in their willingness to use these 2 storage resources, and whether lockers within retailers would increase willingness.

**Objective:**

To determine the willingness of firearm owners and their households to voluntarily and temporarily store firearms with retailers and law enforcement agencies.

**Design, Setting, and Participants:**

In this survey study, a nationally representative sample of US adults was surveyed online in May 2024 cross-sectionally.

**Exposure:**

Living in a home with a firearm.

**Main Outcomes and Measures:**

The willingness to use each storage resource was assessed by participants’ willingness to store in at least 1 of 7 given circumstances. Three multivariable logistic regression models adjusting for participant characteristics were used to examine which characteristics were associated with (1) willingness to store with firearm retailers for any reason, (2) willingness to store with law enforcement agencies for any reason, and (3) whether lockers would make participant more willing to store with retailers.

**Results:**

A sample of 3146 US adults who live in a home with a firearm was surveyed (51.26% male; 95% CI, 49.37%-53.14%). Of participants, 41.38% (95% CI, 39.52%-43.27%) reported willingness to store firearms with retailers and 34.09% (95% CI, 32.31%-35.92%) to store with law enforcement agencies in at least 1 circumstance. The most common circumstance for willingness for both storage entities was concern that someone else in the home would use the firearm to hurt others (firearm retailers, 23.98% [95% CI, 22.41%-25.64%]; law enforcement agencies, 20.05% [95% CI,18.58%-21.61%]). Participants who believed that household firearm access increases suicide risk had increased odds of willingness to store with retailers (adjusted odds ratio, 1.68; 95% CI, 1.41-2.01) and law enforcement agencies (adjusted odds ratio, 1.86; 95% CI 1.55-2.24). Locker installation within retailers increased willingness to store with retailers for 22.24% (95% CI, 20.68%-23.89%) of participants.

**Conclusions and Relevance:**

In this survey study, a meaningful minority of those who live in a home with a firearm reported willingness to temporarily store firearms with retailers and law enforcement agencies in some circumstances. Locker installation within retailers and increased knowledge of the relationship between household firearm access and suicide may increase willingness. Findings provide policymakers with actionable strategies, such as supporting locker installation and reducing liabilities for firearm retailers.

## Key Points

**Question:**

Are firearm owners and others in their households willing to voluntarily and temporarily store firearms with firearm retailers and law enforcement agencies?

**Findings:**

In this survey study with an unweighted sample of 3146 US adults who live in a home with a firearm, 41.38% were willing to store with retailers and 34.09% with law enforcement agencies, with similar demographic patterns and concerns for both storage resources; 22.24% of all participants reported increased willingness to store with retailers if lockers were installed.

**Meaning:**

These findings suggest that decreasing concerns and installing lockers within firearm retailers may increase the willingness of firearm owners to voluntarily, temporarily consider out-of-home storage for their firearms.

## Teaser

This survey study examines the willingness of firearm owners and their households to voluntarily and temporarily store firearms with retailers and law enforcement agencies.

## Introduction

Firearm access is associated with increased risk of suicide,^1,2^ intimate partner homicide,^3^ and unintentional injuries and death.^2^ Firearms are the most lethal mean of suicide and homicide^4,5^ and are used in a majority of suicides and homicides.^6^ When there is heightened concern of harm, including unauthorized firearm access, voluntarily storing firearms outside the home may help decrease risk.^7,8^

Firearm retailers and law enforcement agencies (LEAs) are options for firearm owners seeking voluntary, temporary, out-of-home storage solutions. Firearm owners consistently rate retailers and LEAs as trusted messengers for suicide prevention.^9,10^ In studies of Mountain West states^11^ and Colorado and Washington,^12^ 50% of retailers and 60% to 65% of LEAs had received storage requests in the past year, and 48% of retailers and 53% to 75% of LEAs reported currently providing the service. Both a majority of surveyed retailers and LEAs showed willingness to provide temporary storage, including when owners expressed concerns about adolescents in the home or another adult family member, and when owners faced personal crises.^11^ In a study of retailers in the Colorado Gun Shop Project, 14% of retailers had assisted a customer whom they perceived to be in suicidal crisis with temporary storage.^13^ Some retailers and LEAs have also agreed to be listed on maps of organizations that can provide temporary storage.^14^

Recent federal and state actions have begun addressing some of these challenges faced by retailers and LEAs offering temporary storage.^15,16,17^ The Bureau of Alcohol, Tobacco, Firearms and Explosives recently clarified that retailers can offer lockers for temporary storage,^18^ addressing some liability concerns. In addition, some states, such as Louisiana and Washington,^19,20^ have prohibited retailers from facing liability for firearm injuries and deaths that occur after returning a firearm from temporary storage. Multiple programs also support retailers in providing temporary storage including the Gun Shop Project,^21^ Pause to Protect,^22^ and the Armory Project.^23^

Focus is now needed to understand firearm owners’ willingness to use temporary storage. However, research on firearm owners’ use of and willingness to use temporary storage with retailers and LEAs remains limited and geographically bound. In a survey of firearm owners and their families in Colorado and Washington, 45% reported they would be likely to consider retailers and 39% would be likely to consider LEAs for temporary storage.^24^ Firearm owners and their families’ concerns about temporary storage included privacy, logistics of storage and retrieval, costs, background check requirements, and potential damage or loss.^24,25^ However, those studies did not distinguish between concerns specific to retailers vs LEAs. This distinction matters, as concerns may be unique. For example, LEA leaders report that Black firearm owners and those who distrust the government may be hesitant to temporarily store firearms with them.^16^

In addition, studies have not examined whether lockers within retailers could change firearm owners’ attitudes on temporary storage. Lockers allow firearm owners to rent a secure space, store their firearms independently, and retrieve them at their convenience. Although lockers may address firearm owners’ concerns about privacy and the logistics of storing and retrieving firearms, to our knowledge, there is no research investigating whether lockers would increase willingness to use temporary storage.

In this study, we conducted a nationally representative survey of adults and asked those who live in a home with a firearm about willingness to and concerns about temporarily storing their firearms outside the home, asking separately about firearm retailers and LEAs. In addition, we asked whether installing lockers within retailers would increase participants’ willingness to temporarily store firearms with retailers. Finally, we explored which participant characteristics were associated with willingness to consider temporary storage with retailers and LEAs.

## Methods

### Data

In May 2024, we collected nationally representative data of US adults with Ipsos KnowledgePanel, the largest probability-based online panel of US adults. Ipsos regularly sends their KnowledgePanel participants surveys to complete, and of the 12 822 participants invited to participate, 8647 (67%) opened the survey and 8009 (93%) consented electronically to participate. All participants who consented completed the survey. Final survey weights were calculated by Ipsos using iterative proportional fitting, and weights were raked to geodemographic benchmarks of the adult US population using the 2023 March Supplement of the Current Population Survey (additional information can be found in the eMethods in Supplement 1). This study was approved by the Rutgers University institutional review board. This study followed the American Association for Public Opinion Research (AAPOR) reporting guidelines. Analyses included all participants who reported at least 1 firearm in or around their home.

### Temporary Storage Items

Participants were presented with 2 series of checkboxes (1 for firearm retailers and 1 for LEAs) and asked to indicate whether they would consider storing with the entity in 7 different circumstances. Participants could also check that they would never consider temporary storage. Participants were also presented with 2 series of checkboxes (1 for firearm retailers and 1 for LEAs) and asked to indicate 8 potential concerns they had with temporarily storing firearms with each. Participants could also indicate they had no concerns. Participants were then asked whether retailers installing lockers in which participants could temporarily store their firearms (without having to explain their storage decision or go through a background check) would increase their willingness to temporarily store firearms at a retailer. All temporary storage items can be found in eMethods in Supplement 1.

### Participant Characteristics

#### Demographics

Participants self-reported their gender, race and ethnicity (through a list of options provided by IPSOS [ie, Black, non-Hispanic; Hispanic; White, non-Hispanic; and other or ≥2 races, non-Hispanic]), age, military experience (if they were currently serving or had ever served), and political beliefs. Race and ethnicity were assessed because racial and/or ethnic discrimination may influence whether individuals would be willing to temporarily store firearms with law enforcement.^16^ We also determined whether participants lived in a metropolitan statistical area.

#### Others in the Home

We asked participants whether any children or another adult lived in their home. We also asked whether the participant was concerned that someone else in the home would harm themselves or someone else.

#### Gun Violence Exposure

Participants’ direct gun violence exposure was determined if participants answered positively to either having been threatened with a firearm by another person or having been intentionally shot by another person. Participants’ indirect gun violence exposure was determined if participants answered positively to either personally knowing someone who had been shot on purpose by another person, witnessing a shooting in their neighborhood, or hearing gun shots in their neighborhood. Defensive firearm use was determined if participants answered positively to either telling someone who was threatening them that they had a firearm, showing a firearm to someone who was threatening them, firing a firearm near someone who was threatening them, or firing a firearm at someone who was threatening them.

#### Suicide Exposure

We asked whether participants ever knew someone personally who had died by firearm suicide. We also asked whether participants had ever experienced suicidal ideation by using the Self-Injurious Thoughts and Behaviors Interview–Self-Report version.^26^

#### Beliefs

To measure threat sensitivity, we asked participants 3 items from the Posttraumatic Cognitions Inventory (PTCI; α = 0.89).^27^ Participants were also asked about the relationship between household firearms and suicide risk using a 5-point agreement scale, and we created a binary variable of whether the participant believes that a household firearm increases the risk of suicide. Similarly, participants were asked about the relationship between household firearms and protection from home invasion using a 5-point agreement scale, and collapsed response options into a binary: “Keeping a firearm in or around the home protects during a home invasion.” The original survey items for all beliefs can be found in the eMethods in Supplement 1.

#### Firearms and Arrest History

We asked participants whether they or someone in their home had ever temporarily stored a firearm or part of a firearm outside the home with friends or family. We also asked participants whether they made decisions on how firearms in the home are stored. In addition, we asked participants to select their primary reason for having access to a firearm. We collapsed response options (see eMethods in Supplement 1) into a binary: “Protection at or away from home is the primary reason for firearm access.” Finally, we asked whether participants had ever been arrested.

### Statistical Analysis

All analyses were conducted with weighted data, and results were considered significant at α = .05. We first conducted descriptive statistics with weighted data to examine when participants would consider temporary storage with retailers and LEAs, participants’ concerns about temporary storage with retailers and LEAs, and how lockers would change participants’ willingness to store with retailers. We also used 2-sided χ^2^ tests to determine whether these temporary storage items were associated with whether participants decided how firearms in the home were stored (eTable 1 in Supplement 1). Next, we used 3 multivariable logistic regression models adjusting for participant characteristics to examine which characteristics were associated with (1) willingness to store with firearm retailers for any reason, (2) willingness to store with LEAs for any reason, and (3) whether lockers would make participant more willing to store with retailers. The Little missing completely at random test was conducted, and data were determined to be not missing at random. The variables with the highest weighted percentage of missing data were the 3 outcome variables: whether lockers would change willingness (1.76%), willingness to store with LEAs (1.62%), and willingness to store with retailers (1.44%). For the multivariable analyses, listwise deletion was used in the 3 multivariable analyses for participants with missing data, and a weighted 6% of participants were excluded from analyses. All analyses were conducted in Stata statistical software version 17 (Stata Corp) using the svy set of commands for weighted data.

## Results

We included all participants who reported that they lived with a firearm in or around their home (3146 unweighted participants). Of these participants, 51.26% (95% CI, 49.37%-53.14%) were male, 9.92% (8.82%-11.13%) were Black non-Hispanic, 12.09% (10.78%-13.55%) were Hispanic, 72.56% were White, non-Hispanic, and 5.44% (4.56%-6.48%) were other non-Hispanic (defined as >1 race and any racial identity other than Black or White) (Table 1). Of these participants, 68.12% (95% CI, 66.30%-69.90%) reported they decided how home firearms were stored.

**Table 1.** Characteristics of Survey Respondents Who Live in a Home With a Firearm

Characteristic	Respondents		Missingness, weighted % (95% CI)
	Unweighted No. (N = 3146)	Weighted % (95% CI)	
Demographics			
Gender			
Male	1677	51.26 (49.37-53.14)	0
Female	1469	48.74 (46.86-50.63)	
Race and ethnicity			
Black, non-Hispanic	285	9.92 (8.82-11.13)	0
Hispanic	276	12.09 (10.78-13.55)	
White, non-Hispanic	2391	72.56 (70.72-74.30)	
Other, non-Hispanic^a^	194	5.44 (4.56-6.48)	
Age, y			
18-29	343	17.04 (15.43-18.79)	0
30-44	709	24.06 (22.47-25.73)	
45-59	841	26.11 (24.53-27.76)	
≥60	1253	32.80 (31.14-34.47)	
Lives in metropolitan statistical area	2505	79.75 (78.20-81.21)	0
Active military or veteran	500	13.75 (12.59-14.99)	0.45 (0.22-0.93)
Political beliefs			
Conservative	1426	45.09 (43.22-46.97)	0.73 (0.42-1.28)
Moderate	1143	38.75 (36.90-40.63)	
Liberal	561	16.16 (14.89-17.53)	
Others in the home			
Child(ren) in the home	825	29.10 (27.37-30.90)	0
Other adult in the home	2671	85.51 (84.15-86.77)	0
Concerned someone else in home will harm self or others	69	2.45 (1.88-3.17)	0.43 (0.22-0.84)
Interpersonal gun violence exposure			
Direct gun violence exposure	615	19.19 (17.74-20.72)	0.23 (0.00-0.54)
Indirect gun violence exposure	1808	57.86 (55.99-59.71)	0.53 (0.30-0.93)
Have used guns defensively	256	8.33 (7.31-9.49)	0.57 (0.34-0.96)
Suicide exposure			
Knew someone who died by firearm suicide	1161	34.54 (32.81-36.32)	0.29 (0.14-0.60)
Suicide ideation (lifetime)	533	18.49 (17.0-20.08)	0.45 (0.24-0.84)
Beliefs			
Threat sensitivity, mean (SD)	10.81 (4.3)	11.02 (10.85-11.18)	0.50 (0.31-0.81)
Household firearm increases risk of suicide	1362	43.47 (41.60-45.36)	1.29 (0.92-1.80)
Household firearm protects in home invasion	2641	85.70 (84.37-86.94)	0.44 (0.26-0.75)
Firearms			
Decides how household firearms are stored	2226	68.12 (66.30-69.90)	0.28 (0.11-0.75)
Have stored firearm(s) outside home with family or friends	142	4.55 (3.82-5.41)	0.44 (0.24-0.81)
Protection is primary reason for access	1498	49.29 (47.40-51.18)	0.51 (0.30-0.88)
Other			
Ever arrested	506	16.20 (14.85-17.65)	0.26 (0.13-0.50)

^a^ Includes participants who identified as more than 1 race and those whose racial identity is one other than Black or White.

Regarding willingness, 41.38% (95% CI, 39.52%-43.27%) reported willingness to temporarily store with retailers and 34.09% (95% CI, 32.31%-35.92%) with LEAs in at least 1 situation (Table 2). A majority of participants said they would never store firearms with retailers (58.62% [95% CI, 56.73%-60.48%]) and LEAs (65.91% [95% CI, 64.08%-67.69%]). When asking participants when they would be willing to temporarily store with firearm retailers and LEAs, the most common reason was if the participant was concerned that someone else in the home would use the firearm to hurt others (retailers, 23.98% [95% CI, 22.41%-25.64%]; LEAs, 20.05% [95% CI, 18.58%-21.61%]).

**Table 2.** Considerations and Concerns of Temporarily Storing a Firearm With Firearm Retailers and Law Enforcement (N = 3146)

Variable	With firearm retailers		With law enforcement	
	Weighted % (95% CI)	Missingness, weighted % (95% CI)	Weighted % (95% CI)	Missingness, weighted % (95% CI)
Would consider temporarily storing				
If I am experiencing severe emotional distress	11.75 (10.58-13.03)	1.44 (1.02-2.02)	11.09 (9.95-12.34)	1.62 (1.16-2.28)
If someone else in my home is experiencing severe emotional distress	18.53 (17.09-20.06)		16.16 (14.81-17.61)	
If I was concerned someone in the home would use the firearm to hurt others	23.98 (22.41-25.64)		20.05 (18.58-21.61)	
If I am going on vacation	15.44 (14.10-16.88)		12.25 (11.03-13.58)	
If children or adolescents will be visiting my home	11.03 (9.91-12.26)		9.06 (8.03-10.21)	
If other people will be in my home unsupervised (eg, home renovation)	21.13 (19.63-22.71)		15.2 (13.89-16.60)	
Other	1.27 (0.90-0.18)		1.22 (0.90-1.66)	
I would never do this	58.62 (56.73-60.48)		65.91 (64.08-67.69)	
Concerns with storing				
Concerns that firearm(s) will get lost or damaged	36.01 (34.20-37.86)	1.72 (1.28-2.30)	33.79 (32.02-35.61)	2.05 (1.54-2.74)
Concerns that I will be forced to reveal my reason for requesting this service	10.75 (9.59-12.03)		17.53 (16.13-19.02)	
Concerns that I will have to undergo a background check when I retrieve firearm(s)	11.53 (10.32-12.88)		16.08 (14.71-17.56)	
Cost	34.49 (32.69-36.32)		21.91 (20.37-23.53)	
Proximity of the retailer or police station to my home	27.31 (25.66-29.03)		22.89 (21.35-24.51)	
Concerns that using this service will leave my home unprotected during an emergency	34.75 (32.96-36.59)		33.44 (31.68-35.26)	
Concerns that I will face discrimination when requesting this service or retrieving firearms	8.17 (7.19-9.27)		15.44 (14.11-16.88)	
The firearm(s) I would want to store belongs to someone else in my home	7.61 (6.61-8.74)		7.14 (6.19-8.21)	
Concerns that I will be arrested when requesting this service or retrieving firearms	NA		10.79 (9.61-12.08)	
I do not have any concerns	32.17 (30.42-33.98)		33.12 (31.36-34.93)	
Would lockers increase willingness to temporarily store firearms at a retailer under some circumstances?				
No, I still would not do this under any circumstance	61.13 (59.26-62.97)	1.76 (1.29-2.40)	NA	NA
No, I am always completely willing to do this under some circumstances, so this does not change anything	16.62 (15.27-18.07)		NA	
Yes, this would make me more willing in some circumstances	22.24 (20.68-23.89)		NA	

Abbreviation: NA, not applicable.

Just over one-third of participants were concerned that a firearm would get lost or damaged with retailers (36.01% [95% CI, 34.20%-37.86%]) and LEAs (33.79% [95% CI, 32.02%-35.61%]). Regarding concerns, 15.44% (95% CI, 14.11%-16.88%) were concerned they would face discrimination when using this service with LEAs, compared with 8.17% (95% CI, 7.19%-9.27%) with retailers. Our post hoc analyses found that only 10.79% of those who reported discrimination concerns storing with law enforcement were Black (eTable 2 in Supplement 1). Just over one-fifth (22.24% [95% CI, 20.68%-23.89%]) reported that lockers would make them more willing to store with retailers, whereas 16.62% (95% CI, 15.27%-18.07%) reported that lockers did not change their willingness, and 61.13% (95% CI, 59.26%-62.97%) reported they would still never use retailers for temporary storage.

Table 3 describes participant characteristics associated with willingness for temporary storage. Participants with increased odds of willingness to store with both retailers and LEAs were Black (compared with White) (retailers, adjusted odds ratio [aOR], 1.42 [95% CI, 1.05-1.93]; LEAs, aOR, 1.64 [95% CI, 1.20-2.24]), lived with another adult in the home (retailers, aOR, 1.35 [95% CI, 1.06-1.73]; LEAs, aOR, 1.36 [95% CI, 1.05-1.77]), were concerned someone else in the home would harm themselves or others (retailers, aOR, 2.93 [95% CI, 1.52-5.68]; LEAs, aOR, 2.54 [95% CI, 1.38-4.66]), had used firearms defensively (retailers, aOR, 1.65 [95% CI, 1.20-2.28]; LEAs, aOR, 1.84 [95% CI, 1.32-2.56]), believed that household firearms increases suicide risk (retailers, aOR, 1.68 [95% CI, 1.41-2.01]; LEAs, aOR, 1.86 [95% CI, 1.55-2.24]), and had previously stored firearms outside the home with friends or family (retailers, aOR, 1.82 [95% CI, 1.21-2.74]; LEAs, aOR, 1.56 [95% CI, 1.04-2.35]). Participants with decreased odds of willingness to temporarily store with both retailers and LEAs believed that firearms in the home protect in cases of home invasion (retailers, aOR, 0.65 [95% CI, 0.50-0.83]; LEAs, aOR, 0.63 [95% CI, 0.49-0.81]).

**Table 3.** Multivariable Logistic Regression Results Showing Factors Associated With Willingness to Consider Temporary Firearm Storage, Adjusted for Participant Characteristics

Variable	aOR (95% CI) (N = 3146)		
	Would consider storing with retailers (n = 2967)	Lockers would make more willing to store with retailers (n = 2959)	Would consider storing with law enforcement (n = 2963)
Demographics			
Female gender	1.05 (0.85-1.28)	0.68 (0.54-0.86)	1.25 (1.02-1.55)
Race and ethnicity			
Black, non-Hispanic	1.42 (1.05-1.93)	1.00 (0.69-1.43)	1.64 (1.20-2.24)
Hispanic	1.29 (0.97-1.72)	1.03 (0.74-1.42)	1.39 (1.03-1.87)
White, non-Hispanic	1 [Reference]	1 [Reference]	1 [Reference]
Other, non-Hispanic^a^	0.92 (0.61-1.39)	1.00 (0.60-1.68)	1.27 (0.83-1.96)
Age, y			
18-29	1 [Reference]	1 [Reference]	1 [Reference]
30-44	0.83 (0.61-1.12)	0.74 (0.53-1.03)	0.86 (0.63-1.18)
45-59	0.68 (0.51-0.93)	0.59 (0.43-0.84)	0.84 (0.61-1.14)
≥60	0.59 (0.43-0.80)	0.52 (0.37-0.74)	0.85 (0.62-1.16)
Lives in metropolitan area	1.11 (0.90-1.37)	1.10 (0.85-1.42)	1.21 (0.97-1.51)
Active military or veteran	0.98 (0.76-1.25)	0.81 (0.60-1.09)	0.90 (0.70-1.16)
Political beliefs			
Conservative	1 [Reference]	1 [Reference]	1 [Reference]
Moderate	1.07 (0.88-1.29)	1.22 (0.96-1.54)	1.04 (0.85-1.27)
Liberal	1.38 (1.08-1.77)	1.43 (1.07-1.90)	1.08 (0.83-1.39)
Others in the home			
Child(ren) in the home	0.82 (0.66-1.01)	1.05 (0.83-1.34)	0.85 (0.69-1.07)
Other adult in the home	1.35 (1.06-1.73)	1.43 (1.06-1.94)	1.36 (1.05-1.77)
Concerned someone in home will harm self or others	2.93 (1.52-5.68)	3.14 (1.72-5.71)	2.54 (1.38-4.66)
Interpersonal gun violence exposure			
Direct gun violence exposure	0.94 (0.74-1.19)	1.06 (0.81-1.38)	0.84 (0.66-1.07)
Indirect gun violence exposure	1.24 (1.04-1.48)	1.15 (0.93-1.41)	1.07 (0.89-1.29)
Have used guns defensively	1.65 (1.20-2.28)	1.33 (0.93-1.92)	1.84 (1.32-2.56)
Suicide exposure			
Knew someone who died by firearm suicide	1.06 (0.89-1.26)	1.05 (0.86-1.31)	1.05 (0.87-1.26)
Suicide ideation (lifetime)	1.32 (1.04-1.68)	1.27 (0.96-1.66)	1.18 (0.92-1.51)
Beliefs			
Threat sensitivity	0.99 (0.97-1.01)	0.98 (0.96-1.01)	0.99 (0.96-1.01)
Firearm increases risk of suicide	1.68 (1.41-2.01)	1.66 (1.34-2.06)	1.86 (1.55-2.24)
Firearm protects in home invasion	0.65 (0.50-0.83)	0.75 (0.57-1.00)	0.63 (0.49-0.81)
Firearms			
Decides how household firearms are stored	1.02 (0.82-1.27)	1.08 (0.83-1.39)	0.87 (0.70-1.09)
Have stored firearm(s) outside home with family or friends	1.82 (1.21-2.74)	1.80 (1.17-2.78)	1.56 (1.04-2.35)
Protection is primary reason for access	1.04 (0.87-1.24)	1.10 (0.89-1.36)	1.09 (0.91-1.32)
Other			
Ever arrested	0.87 (0.69-1.11)	0.89 (0.67-1.18)	0.65 (0.50-0.84)

Abbreviation: aOR, adjusted odds ratio.

^a^ Includes participants who identified as more than 1 race and those whose racial identity is one other than Black or White.

Some characteristics were significantly associated with only 1 storage entity. Participants describing themselves as liberal (compared with conservative) (aOR, 1.38 [95% CI, 1.08-1.77]), those who had indirectly experienced gun violence (aOR, 1.24 [95% CI, 1.04-1.48]), and those who had experienced suicidal ideation in their lifetime (aOR, 1.32 [95% CI, 1.04-1.68]) had increased odds of willingness to store with retailers. Participants who were older had decreased odds of willingness to store with retailers (aged 45-59 years, aOR, 0.68 [95% CI, 0.51-0.93]; aged ≥60 years, aOR, 0.59 [95% CI, 0.43-0.80]), and participants who had ever been arrested had decreased odds of willingness to store with LEAs (aOR, 0.65 [95% CI, 0.50-0.84]). Female participants had increased odds (aOR, 1.25 [95% CI, 1.02-1.55]) of willingness to store with LEAs.

## Discussion

In this survey study, we characterized both willingness and obstacles to temporarily storing firearms with retailers and LEAs among a national sample of US adults with household firearm access. In addition, we examined whether installing lockers at retailers might influence individuals’ willingness to temporarily store with retailers. Three primary results emerged. First, a meaningful minority reported willingness to temporarily store firearms with firearm retailers (41.38%) and LEAs (34.09%), with similar demographic patterns and barriers reported for both options. Second, willingness to temporarily store firearms outside the home was more commonly endorsed in response to concerns for others than for oneself. Finally, lockers appear to have the potential to increase willingness to use temporary firearm storage at retailers.

There were several unexpected findings, including that Black adults had greater odds of willingness to temporarily store with LEAs. This was unexpected, as 15.44% of all adults were concerned that they would face discrimination when requesting temporary storage or retrieving firearms from LEAs, and a qualitative study found that Black adults may be less willing to store with LEAs.^16^ Our post hoc analyses found that only 10.79% of those who reported discrimination concerns storing with law enforcement were Black (eTable 2 in Supplement 1). As new firearm owners since 2019 were more likely to be Black, Hispanic, and female,^28^ it is possible that these new firearm owners are more open to temporary firearm storage and this is why Black participants had increased odds of willingness to store with LEAs. Two other unexpected findings were that threat sensitivity and home protection as the reason for having a firearm were not associated with willingness to store with either retailers or LEAs.^29,30,31^ It may be that these characteristics promote acquiring firearms but have less relevance to how firearms should be managed during a crisis.

Characteristics associated with willingness for temporary storage with both retailers and LEAs largely correspond with an understanding of household firearm risk. Those living with other adults, those concerned someone in the home may hurt themselves or others, those who have used firearms defensively, those who believe firearm access increases suicide risk, and those who have previously stored firearms with family or friends expressed greater willingness to utilize temporary storage with both storage providers. In contrast, those who believed firearms provide protection during home invasions were less willing to utilize temporary storage with either storage entity.

The most reported obstacles to storage across entities related to cost, firearms being lost or damaged, and firearms not being accessible in emergencies. Such obstacles highlight the importance of addressing logistical barriers and emphasizing the transient nature of this option (eg, acute suicidal crisis). Importantly, a majority of participants selected “I would never do this,” when asked about retailers (58.62%) and LEAs (65.91%). Retailers and police officers can engage firearm owners in conversations about temporary storage options even outside of crisis situations to normalize this practice, explain the many reasons for temporary storage, and address common concerns.

Across storage providers, willingness was more commonly endorsed within the context of perceived risk for others compared with concerns for self. This aligns with prior work that found that firearm owners were more open to adopting secure storage practices to prevent someone else’s suicide than to prevent their own suicide^32^ and indicates that public health efforts to promote temporary storage should emphasize how such practices can protect loved ones.

Our results also highlight that lockers at retailers could potentially have a meaningful effect. Nearly one-quarter of participants (22.24%) indicated that they would be more willing to store firearms at a retailer if lockers were installed. Bureau of Alcohol, Tobacco, Firearms and Explosives guidelines and state laws such as those in Louisiana and Washington demonstrate momentum toward addressing concerns of firearm retailers. However, large-scale implementation of lockers will require financial investment. Firearm retailers engaging with existing resources (eg, Pause to Protect or the Armory Project)^22,23^ may further encourage such efforts. However, similar percentages of participants indicated that they would never use retailer storage regardless of locker availability, suggesting that lockers may only increase willingness among those already open to using storing with retailers.

### Limitations

There are several limitations, including the small but nonrandom nature of missing data. The items with the most missing data were how lockers would change willingness (1.76%), willingness to store with LEAs (1.62%), and willingness to store with retailers (1.44%). Some participants may have felt uncomfortable discussing temporary storage. These participants may be less willing to use temporary storage, which would have decreased overall participant willingness had they responded to these items. In addition, cross-sectional self-reporting of data precludes causality and introduces the possibility of biases in response patterns. Furthermore, although we examined how a participants’ firearm storage decision-making ability was associated with willingness for temporary storage, we were not able to examine how firearm ownership was associated.

## Conclusions

These findings advance our understanding of willingness of US adults to temporarily store their firearms outside the home and the obstacles preventing broad use of these services. Additional research should examine the feasibility of installing lockers at retailers and whether lockers lead to an uptake of temporary storage. Researchers should also examine cultural factors influencing temporary storage, including how discrimination might affect minoritized groups’ access to and perception of this resource. Temporary, voluntary out-of-home firearm storage with retailers and LEAs are supported by firearm owners when firearm injury risk exists, and paths toward increasing this intervention should be prioritized.

## Notes

Supplement 1.
eMethods
**eTable 1.** Considerations and concerns of temporarily storing a firearm with firearm retailers and law enforcement, by whether participant decides household firearm storage (N=3,018) **eTable 2.** Demographic characteristics of participants who indicated that they were concerned about experiencing discrimination when temporarily storing firearms or retrieving firearms

Supplement 2.
Data Sharing Statement
