# Supplementary material for: Owner Willingness to Temporarily Store Firearms With Firearm Retailers and Law Enforcement Agencies
Source: JAMA Netw Open. 2025 Jul 16;8(7):e2521921. doi: 10.1001/jamanetworkopen.2025.21921 (PMC12268481; doi:10.1001/jamanetworkopen.2025.21921)
Supplement: Supplement 1. — eMethods eTable 1. Considerations and concerns of temporarily storing a firearm with firearm retailers and law enforcement, by whether participant decides household firearm storage (N=3,018) eTable 2. Demographic characteristics of participants who indicated that they were concerned about experiencing discrimination when temporarily storing firearms or retrieving firearms [file jamanetwopen-e2521921-s001.pdf]

Supplemental Online Content

Paruk J, Wright-Kelly E, Barnard L, Anestis M. Owner willingness to temporarily store firearms with firearm retailers and law enforcement agencies. *JAMA Netw Open*. 2025;8(7):e2521921. doi:10.1001/jamanetworkopen.2025.21921

eMethods

eTable 1. Considerations and concerns of temporarily storing a firearm with firearm retailers and law enforcement, by whether participant decides household firearm storage (N=3,018)

eTable 2. Demographic characteristics of participants who indicated that they were concerned about experiencing discrimination when temporarily storing firearms or retrieving firearms

This supplemental material has been provided by the authors to give readers additional information about their work.

© 2025 Paruk J et al. *JAMA Network Open*.

eMethods

Data Description

Data was collected with Ipsos Public Affairs (Ipsos) on Knowledge Panel (KP). KP uses probability-based sampling for recruitment to provide a sampling frame that is representative of U.S. adults. KP households are provided with internet access and hardware if needed. When households are recruited for a survey, they are notified by email. KP panel members complete an average of three to four surveys per month. KP participants are included in raffles and sweepstakes with cash rewards and other prizes for completing surveys. For this survey, Ipsos invited one adult from a representative sample of KP households to complete the survey. Data were collected between May 15, 2024 and May 28, 2024.

The design weights for participants in this study were computed to reflect their selection probabilities. Weights were raked to the geographic distributions to the U.S. adult population using benchmarks from the 2023 March Supplement of the Current Population Survey (gender, race/ethnicity, census region, education, household income). Weights were scaled to add up to the total number of respondents.

Temporary Storage Items

If a local firearm retailer offered on-site temporary firearm storage, when would you consider storing one or more firearms from in/around your home with them? Select all that apply.

1. If I am experiencing severe emotional distress
2. If someone else in my home is experiencing severe emotional distress
3. If I was concerned someone in the home would use the firearm to hurt others
4. If I am going on vacation
5. If children or adolescents will be visiting my home
6. If other people will be in my home unsupervised (e.g. during home renovation)
7. Other (please specify)
8. I would never do this

If local police offered on-site temporary firearm storage, when would you consider storing one or more firearms from in/around your home with them? Select all that apply.

1. If I am experiencing severe emotional distress
2. If someone else in my home is experiencing severe emotional distress
3. If I was concerned someone in the home would use the firearm to hurt others
4. If I am going on vacation
5. If children or adolescents will be visiting my home
6. If other people will be in my home unsupervised (e.g. during home renovation)
7. Other (please specify)
8. I would never do this

Which of the following factors are concerns to storing firearm(s) from in/around your home temporarily with a firearm retailer? Select all that apply.

1. Concerns that firearm(s) will get lost or damaged
2. Concerns that I will be forced to reveal my reason for requesting this service

© 2025 Paruk J et al. *JAMA Network Open*.

3. Concerns that I will have to undergo a background check when I am ready to retrieve the firearm(s)
4. Cost
5. Proximity of the retailer to my home
6. Concerns that using this service will leave my home unprotected during an emergency
7. Concerns that I will face discrimination when requesting this service or when I retrieve the firearms
8. The firearm(s) I would want to store belongs to someone else in my home/the firearms don't belong to me
9. I do not have any concerns

Which of the following factors are concerns to storing your firearm(s) temporarily with local police? Select all that apply.
1. Concerns that firearm(s) will get lost or damaged
2. Concerns that I will be forced to reveal my reason for requesting this service
3. Concerns that I will have to undergo a background check when I am ready to retrieve the firearm(s)
4. Cost
5. Proximity of the police station to my home
6. Concerns that using this service will leave my home unprotected during an emergency
7. Concerns that I will face discrimination when requesting this service or when I retrieve the firearms
8. Concerns that I will be arrested when requesting this service or when I retrieve the firearms
9. The firearm(s) I would want to store belong to someone else in my home
10. I do not have any concerns

Imagine if a firearm retailer installed lockers in their store and you could store your firearm in a rented locker without having to explain why you are storing the firearm there or go through a background check to retrieve your firearm. Would this increase your willingness to temporarily store your firearm(s) at a local retailer under some circumstances?
0. No, I still would not do this under any circumstances
1. No, I am always completely willing to do this under some circumstances, so this does not change anything
2. Yes, this would make me more willing to store my firearms at a retailer under at least some circumstances

Posttraumatic Cognitions Inventory Items

1. People can't be trusted
2. I can't rely on other people
3. People are not what they seem

Response options:
1. Totally disagree
2. Disagree very much
3. Disagree slightly

© 2025 Paruk J et al. *JAMA Network Open*.

4. Neutral
5. Agree slightly
6. Agree very much
7. Totally agree

The three items were summed to create a continuous variable.

Believes that a household firearm increases the risk of suicide

Please select the answer that you believe best reflects the truth.
1. Keeping a firearm in or around the home is extremely helpful in preventing suicide
2. Keeping a firearm in or around the home is somewhat helpful in preventing suicide
3. Keeping a firearm in or around the home has no impact on the risk for suicide
4. Keeping a firearm in or around the home somewhat increases the risk of suicide
5. Keeping a firearm in or around the home dramatically increases the risk of suicide

Believes that a household firearm protects people during a home invasion

Please select the answer that you believe best reflects the truth.
0. Keeping a firearm in or around the home is extremely helpful in protecting people during a home invasion
1. Keeping a firearm in or around the home is somewhat helpful in protecting people during a home invasion
2. Keeping a firearm in or around the home has no impact on whether people are protected during a home invasion
3. Keeping a firearm in or around the home somewhat increases the risk of harm during a home invasion
4. Keeping a firearm in or around the home dramatically increases the risk of harm during a home invasion

Primary reason for keeping a firearm in or around your home

Which of the following is the primary reason for keeping a firearm in or around your home?
1. Protection at or away from home
2. For hunting, recreation, or sport
3. The firearm(s) belongs to someone else who lives with me
4. Other (please specify)

© 2025 Paruk J et al. *JAMA Network Open*.

eTable 1. Considerations and concerns of temporarily storing a firearm with firearm retailers and law enforcement, by whether participant decides household firearm storage (N=3,018)

	With firearm retailers			With law enforcement		
	Does not decide household firearm storage Weighted % (95% CI)	Decides household firearm storage Weighted % (95% CI)	p-value	Does not decide household firearm storage Weighted % (95% CI)	Decides household firearm storage Weighted % (95% CI)	p-value
**Would consider temporarily storing**						
If I am experiencing severe emotional distress	13.08 (10.89, 15.63)	11.16 (9.82, 12.66)	0.16	12.69 (10.57, 15.16)	10.38 (9.08, 11.85)	0.08
If someone else in my home is experiencing severe emotional distress	22.06 (19.31, 25.09)	16.93 (15.29, 18.70)	0.002	19.36 (16.80, 22.21)	14.74 (13.19, 16.43)	0.003
If I was concerned someone in the home would use the firearm to hurt others	26.04 (23.12, 29.19)	22.92 (21.08, 24.86)	0.08	25.61 (22.69, 28.77)	17.38 (15.75, 19.13)	0.000
If I am going on vacation	13.78 (11.49, 16.45)	16.09 (14.50, 17.82)	0.14	11.15 (9.10, 13.58)	12.56 (11.12, 14.16)	0.32
If children or adolescents will be visiting my home	13.03 (10.88, 15.54)	10.12 (8.86, 11.54)	0.03	10.45 (8.51, 12.77)	8.45 (7.28, 9.79)	0.10
If other people will be in my home unsupervised (e.g. home renovation)	22.36 (19.59, 25.39)	20.45 (18.71, 22.30)	0.27	17.63 (15.10, 20.47)	13.95 (12.49, 15.54)	0.01
Other	2.36 (1.47, 3.77)	0.77 (0.46, 1.27)	0.001	2.18 (1.42, 3.34)	0.79 (0.51, 1.21)	0.001
I would never do this	54.87 (51.36, 58.33)	60.43 (58.20, 62.61)	0.008	58.55 (55.06, 61.97)	69.43 (67.30, 71.47)	0.000
**Concerns with storing**						
Concerns that firearm(s) will get lost or damaged	27.96 (24.93, 31.20)	39.80 (37.60, 42.04)	0.000	26.04 (23.06, 29.25)	37.44 (35.28, 39.65)	0.000
Concerns that I will be forced to reveal my reason for requesting this service	7.27 (5.65, 9.31)	12.36 (10.87, 14.01)	0.000	11.97 (9.84, 14.49)	20.11 (18.36, 21.98)	0.000
Concerns that I will have to undergo a background check when I retrieve firearm(s)	6.97 (5.27, 9.16)	13.47 (11.94, 15.16)	0.000	11.13 (9.06, 13.59)	18.39 (16.67, 20.24)	0.000
Cost	30.98 (27.79, 34.36)	36.17 (34.03, 38.37)	0.01	18.85 (16.20, 21.81)	23.36 (21.49, 25.33)	0.01
Proximity of the retailer to my home	20.92 (18.22, 23.91)	30.31 (28.28, 32.42)	0.000	18.80 (16.23, 21.67)	24.82 (22.93, 26.80)	0.001
Concerns that using this service will leave my home unprotected during an emergency	30.45 (27.30, 33.80)	36.63 (34.48, 38.84)	0.003	27.85 (24.78, 31.13)	35.92 (33.80, 38.10)	0.000
Concerns that I will face discrimination when requesting this service or retrieving firearms	5.53 (4.16, 7.33)	9.41 (8.16, 10.82)	0.001	11.30 (9.17, 13.45)	17.47 (15.79, 19.30)	0.000
The firearm(s) I would want to store belongs to someone else in my home	17.90 (15.33, 20.80)	2.71 (2.06, 3.55)	0.000	17.39 (14.87, 20.22)	2.45 (1.83, 3.27)	0.000
Concerns that I will be arrested when requesting this service or retrieving firearms				7.98 (6.19, 10.23)	12.11 (10.64, 13.74)	0.003
I do not have any concerns	34.08 (30.79, 37.54)	31.33 (29.29, 33.45)	0.17	36.02 (32.70, 39.49)	31.80 (29.76, 33.91)	0.04
**Would lockers increase willingness to temporarily store firearms at a retailer under some circumstances?**			0.001			

© 2025 Paruk J et al. *JAMA Network Open*.

No, I still would not do this under any circumstance	57.23 (53.71, 60.67)	63.0 (60.79, 65.15)				
No, I am always completely willing to do this under some circumstances, so this does not change anything	20.48 (17.79, 23.47)	14.89 (13.39, 16.53)				
Yes, this would make me more willing in some circumstances	22.29 (19.50, 25.35)	22.11 (20.26, 24.08)				

© 2025 Paruk J et al. *JAMA Network Open*.

eTable 2. Demographic characteristics of participants who indicated that they were concerned about experiencing discrimination when temporarily storing firearms or retrieving firearms

	**With retailer**<br>**Weighted N=242**<br>**Weighted N, % (95% CI)**	**With law enforcement**<br>**Weighted N=456**<br>**Weighted N, % (95% CI)**
Gender		
Male	142 (58.73%); (52.05, 65.09)	271 (59.34%); (54.46, 64.03)
Female	100 (41.27%); (34.91, 47.95)	186 (40.66%); (35.97, 45.54)
Race/ethnic identity		
Black, non-Hispanic	25 (10.45%); (7.09, 15.13)	49 (10.79%); (8.17, 14.12)
Hispanic	29 (12.07%); (7.86, 18.08)	53 (11.63%); (8.44, 15.82)
White, non-Hispanic	177 (72.95%); (66.41, 78.62)	327 (71.57%); (66.60, 76.06)
Other, non-Hispanic[a]	11 (4.53%); (2.63; 7.72)	27 (6.02%); (3.75, 9.52)
Age		
18-29	47 (19.37%); (14.18, 25.89)	104 (22.78%); (18.38, 27.86)
30-44	80 (33.09%); (27.15, 39.62)	139 (30.37%); (26.11, 34.98)
45-59	56 (23.30%); (18.31, 29.15)	105 (22.90%); (19.25, 27.02)
60+	59 (24.24%); (19.35, 29.91)	109 (23.95%); (20.38, 27.93)
Lives in metropolitan statistical area	185 (76.24%); (70.19, 81.83)	365 (79.95%), (75.84, 83.51)
Military/veteran	38 (15.90%); (11.70, 21.25)	73 (15.94%); (12.85, 19.60)
Political beliefs		
Conservative	130 (53.54%); (46.88, 60.08)	199 (43.65%); (38.92, 48.50)
Moderate	84 (34.57%); (28.45, 41.25)	164 (36.09%); (31.39, 41.08)
Liberal	29 (11.89%); (8.19, 16.95)	92 (20.26%); (16.72, 24.32)

© 2025 Paruk J et al. *JAMA Network Open*.
