# Supplementary material for: Owner Willingness to Temporarily Store Firearms With Firearm Retailers and Law Enforcement Agencies
Source: JAMA Netw Open. 2025 Jul 16;8(7):e2521921. doi: 10.1001/jamanetworkopen.2025.21921 (PMC12268481; doi:10.1001/jamanetworkopen.2025.21921)
Supplement: Supplement 2. — Data Sharing Statement [file jamanetwopen-e2521921-s002.pdf]

Data Sharing Statement

Paruk. Owner Willingness to Temporarily Store Firearms With Firearm Retailers and Law Enforcement Agencies. *JAMA Netw Open*. Published July 16, 2025.
doi:10.1001/jamanetworkopen.2025.21921

Data
Data available: Yes
Data types: Other (please specify)
Additional Information: The corresponding author will respond to requests for data, either by providing the requester with results and output or with specific deidentified components of the dataset that address the request.
How to access data: mda141@sph.rutgers.edu
When available: With publication

Supporting Documents
Document types: None

Additional Information
Who can access the data: Anyone requesting the data
Types of analyses: For clarification of results or requests regarding the nature of the sample.
Mechanisms of data availability: With investigator support
Any additional restrictions: Investigative team will review the nature of the request to ensure it is appropriate. The full dataset will not be provided for secondary analysis unless the investigative team has approved such a request and, in such circumstances, only the approved analyses will be allowed and the relevant data will be provided.
